# Is new always better: comparison of the femoral neck system and the dynamic hip screw in the treatment of femoral neck fractures

**DOI:** 10.1007/s00402-022-04551-w

**Published:** 2022-07-22

**Authors:** Konrad Schuetze, Jakob Burkhardt, Carlos Pankratz, Alexander Eickhoff, Alexander Boehringer, Christina Degenhart, Florian Gebhard, Raffael Cintean

**Affiliations:** Department of Trauma-, Hand-, and Reconstructive Surgery, Ulm University, Albert-Einstein-Allee 23, 89081 Ulm, Germany

**Keywords:** Hip fracture, Cut out, Complications

## Abstract

**Background:**

Hip fractures in the elderly population are common and the number of patients is rising. For young and geriatric patients with undisplaced fractures osteosynthesis is the primary type of treatment. The dynamic hip screw (DHS) is around for many years and proved its value especially in displaced fractures. Since 2018 the femoral neck system (FNS) is available as an alternative showing promising biomechanical results. The aim of this study is to evaluate clinical results of the FNS and compare it to the DHS.

**Materials and methods:**

Patients older than 18 years with Garden I–IV fractures that were treated with osteosynthesis in a level 1 trauma center were included in the study. Between January 2015 and March 2021, all patients treated with FNS (1-hole plate, DePuy-Synthes, Zuchwil, Switzerland) or DHS (2-hole plate, DePuy-Synthes, Zuchwil, Switzerland) for proximal femur fractures were included in the study. Closed reduction was achieved using a traction table. All operations were carried out by experienced orthopedic trauma surgeons. Primary outcome measures were rate of implant failure (cut out) and surgical complications (hematoma, infection). Secondary outcome measures were Hb-difference, length of hospital stay and mortality.

**Results:**

Overall, 221 patients were included in the study. 113 were treated with FNS, 108 with DHS. Mean age was 69 ± 14 years. There were 17.2% Garden I, 47.5% Garden II, 26.7% Garden III and 8.6% Garden IV fractures. No difference between the groups for age, body mass index (BMI), Charlson comorbidity index (CCI), time to surgery, Pauwels and Garden classification, rate of optimal blade position or tip apex distance was found. FNS showed lower pre- to postoperative Hb-difference (1.4 ± 1.1 g/l vs. 2.1 ± 1.4 g/l; *p* < 0.05), shorter operating time (36.3 ± 11.6 min vs. 54.7 ± 17.4 min; *p* < 0.05) and hospital stay (8.8 ± 4.3 d vs. 11.2 ± 6.8 d; *p* < 0.05). Surgical complications (FNS 13.3% vs. DHS 18.4%, *p* > 0.05), rate of cut out (FNS 12.4% vs. DHS 10.2%, *p* > 0.05) and mortality (FNS 3.5%; DHS 0.9%; *p* > 0.05) showed no difference between the groups. Logistic regression showed that poor blade position was the only significant predictor for cut out and increased the risk by factor 7. Implant related infection (*n* = 3) and hematoma/seroma (*n* = 6) that needed revision was only seen in DHS group.

**Conclusion:**

FNS proved to be as reliable as DHS in all patients with hip fractures. Not the type of implant but blade positioning is still key to prevent implant failure. Still due to minimal invasive approach implant related infections and postoperative hematomas might have been prevented using the FNS.

## Background/introduction

The number of hip fracture patients is predicted to increase and will range between 7.3 and 21.3 million by the year 2050 [1]. In Germany, for example cases increased by 23% from 2009 to 2019 with a ratio of 18/82 for under and over 70 year old patients [2]. While young patient suffer from high energy trauma like traffic accidents, hip fractures in older patients are mainly caused by ground level falls [3]. Surgical treatment options are osteosynthesis, arthroplasty and rarely conservative treatment [4]. Despite modern implants complication rate and mortality is still high [5–7]. Most commonly used implants are three cancellous compression screws (CCS) and the dynamic hip screw (DHS) which showed better results in dislocated and lateral fractures and lesser rate of implant removal [8]. Since 2018 the femoral neck system (FNS; DePuy-Synthes, Zuchwil, Switzerland) was introduced showing promising biomechanical results [9, 10]. Comparative studies evaluating the clinical outcome are still limited. The aim of this study was to evaluate the outcome of patients of all ages treated with the femoral neck system and compare it to patients treated with the dynamic hip screw.

## Methods

Institutional and prior ethical committee approval for the use of data in this study was obtained.

All patients over 18 years with a hip fracture type Garden I–IV that were treated in a level 1 trauma center between January 2015 and March 2021 were retrospectively reviewed. Exclusion criteria were patients with pathologic fractures and patients treated with arthroplasty. Osteosynthesis was used mainly in patients under 65 years following current guidelines [4]. In patients over 65 osteosynthesis was used in Garden I and II fractures and also in a low number of cases in Garden III or IV fractures if the patient was in poor general condition. Until July 2018 only DHS-Blades were used. Starting August 2018 DHS was replaced with the femoral neck system. A traction table was used to achieve closed reduction. None of the cases needed open reduction. FNS and DHS was implanted following AO principles through six different experienced trauma surgeons all of them using both implants. The majority of the patient were mobilized on the first postoperative day with full weight bearing. Weight bearing was only restricted in a few young patients with Garden III and IV fractures that had the capability of partial weight bearing. Postoperative X-rays anterior–posterior and lateral were performed postoperative, after 14 days, 6 weeks and 3 months.

All fractures were classified using the AO/OTA Fracture and Dislocation classification, Garden and Pauwels classification. Blade positioning and tip apex distance was measured by an independent observer in the intraoperative X-rays which were performed in perfect ap and lateral view. For signs of implant failure (cut out) all postoperative X-rays were reviewed. In the 3 month controls the shortening of the femoral neck was measured. Patient charts were reviewed to detect further surgical complications like implant related infections or hematoma/seroma which needed revision surgery.

Primary outcome measures were implant failure and surgical complications. Secondary outcome measures were hemoglobin difference pre- to postoperative and length of hospital stay. Data analysis was performed with IBM SPSS Statistics (V21.0) and Microsoft Excel (V16.3). Demographic characteristics are described as mean and standard deviation. For the primary outcome measures, logistic regression was performed considering all variables related implant failure and surgical complications.

## Results

### Patient population

For 221 patients, medical records were reviewed. Out of these 221 patients, 109 were male and 112 were female. The youngest patient was 20, while the oldest was 98 years old. The mean age was 69.5 ± 15.4 years and did not differ between the groups (DHS 68.5 ± 15.1; FNS 66.6 ± 21.5; *p* > 0.05). 19 patients were preoperatively classified as ASA I, 46 as ASA II, 127 as ASA III and 29 as ASA IV. The DHS was used in 108 cases, FNS in 113 cases. Mean follow-up was 13 months. Time to surgery was approximately 14 h for both groups. There was no significant difference between the groups for age, sex, BMI, ASA classification, Charlson comorbidity index or time to surgery.

### Fracture classification

All fractures were classified according to the AO classification. There was a significant difference within the groups with twice the number of patients with 31-B3 fractures in the DHS group. There were slightly more patients that were treated for dislocated Garden III and IV fractures in FNS group. Using Pauwels classification there were 39 Grade III fractures in DHS group and 40 in FNS group. There was no difference between the groups for Pauwels and Garden classification. Detailed classification is shown in Table 1.

**Table 1:** Patient data for both groups

Variable	DHS	FNS	*p* value
Patients	108	113	
Age	68.5 ± 15.1	70.6 ± 14.9	*p* > 0.05
Sex	m: 50.9 versus w: 49.1%	m: 47.8% versus w: 52.2%	*p* > 0.05
BMI	24.0 ± 5.4	23.1 ± 2.5	*p* > 0.05
CCI	1.29 ± 1.5	1.65 ± 1.7	*p* > 0.05
AO classification			***p* < 0.05**
31-B1	28	41	
31-B2	58	61	
31-B3	22	11	
Garden classification			*p* = 0.617
I	22	16	
II	49	56	
III	29	30	
IV	8	11	
Pauwels classification			*p* = 0.229
I	11	5	
II	58	68	
III	39	40	
Time to surgery [h]	14.8 ± 19.2	13.5 ± 15.9	*p* = 0.684
OP time [min]	54.7 ± 17.4 min	36.3 ± 11.6 min	***p* < 0.05**
Hospital stay [days]	11.3 ± 6.8	8.9 ± 4.3	***p* < 0.05**
Hb-difference [g/dl]	2.1 ± 1.4	1.4 ± 1.2	***p* < 0.05**
Optimal blade position	89.8%	87.6%	*p* = 0.634
Tip apex [mm]	24.3 ± 8.4	23.0 ± 7.8	*p* = 0.235
Surgical complications	18.4%	13.3%	*p* = 0.479
Implant failure	10.2%	12.4%	*p* = 0.605
Hematoma	5.5%	0.9%	
Implant related infection	2.7%	0%	
Femoral neck shortening [mm]	4.8 ± 2.1	5.3 ± 1.9	*p* = 0.455
Shortening > 5 mm	27.7%	27.4%	*p* = 0.621

*p* < 0.05 was used as cut off for bold significance

### Perioperative factors

There was a significant difference between the groups for operating time and Hb-difference. Operating time was approximately 18 min shorter and Hb-difference 0.7 g/dl lower in FNS group. The rate of optimal blade position (center–center und inferior–center) and tip apex distance showed no difference between the groups. Center–center or inferior–center position could be achieved in more than 87% of the cases. Mean tip apex distance was less than 25 mm in both groups.

### Cut out and surgical complications

**Fig. 1:**
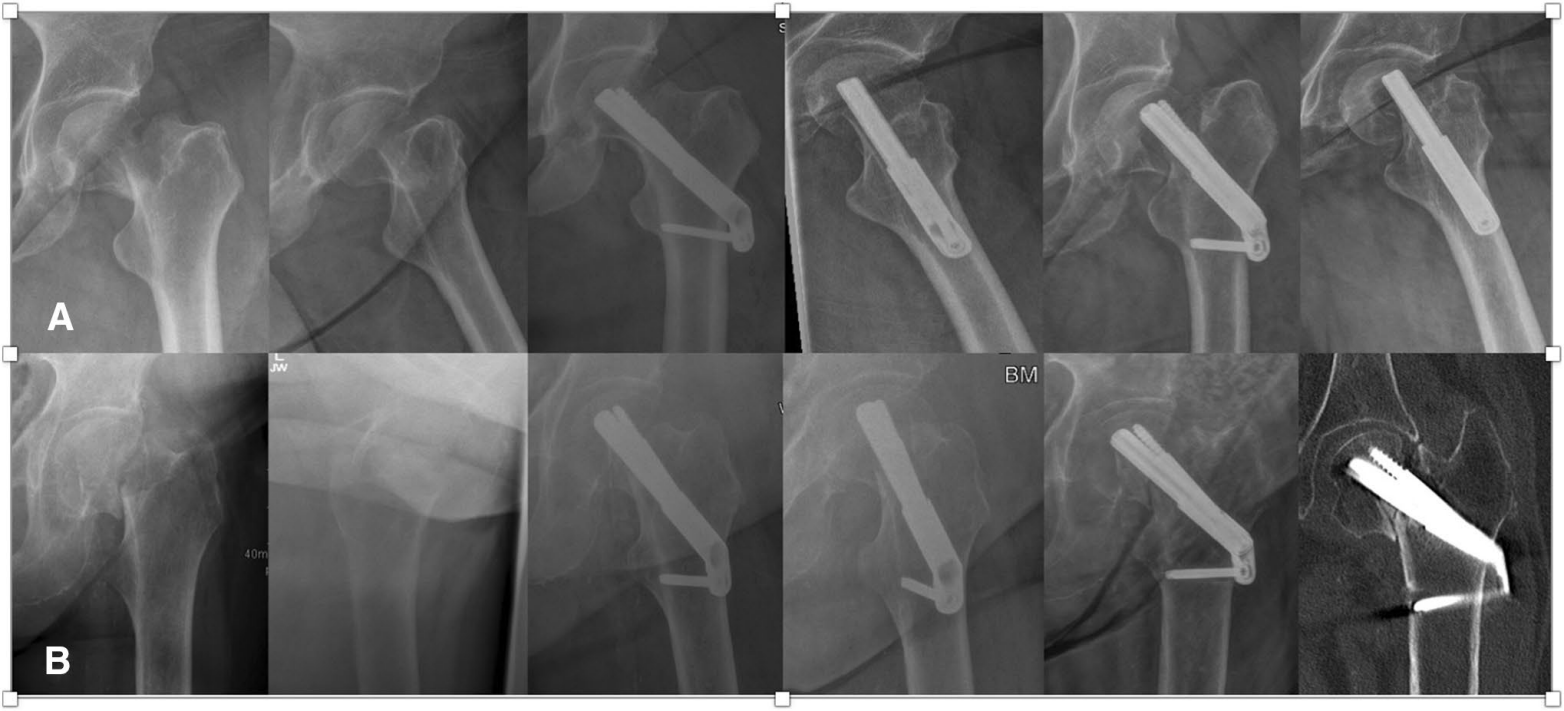
Two cases of cut out: **A** A 55-year-old patient with cut out in the femoral head after 2 month; **B** A 74-year-old patient with implant failure at the 1-hole plate after 6 month

Femoral neck compression showed no difference between the groups and exceeded 5 mm in 27.6% of all cases. The rate of surgical complications showed no significant difference between the groups. In the DHS group there were 11 cut outs, 3 implant related infections and 6 patients with hematoma. All infections were successfully treated with multiple local debridement and antibiotics keeping the implant. In FNS group there were 14 cut outs and 1 hematoma. Implant failure occurred 12 times at the side of the blade and 2 time at the 1-hole plate (Fig. 1). Both implants allowed a comparable mean femoral neck shortening of 5 mm over 3 months. Logistic regression showed that the only significant factor for cut out in both groups was blade positioning. If the blade was not positioned center–center or inferior–center, the risk of cut out increased by factor 7. In the FNS group cut out occurred significantly more often in Pauwels III fracture (*p* < 0.05). High age was no significant predictor of implant failure. Overall 52 patients older than 60 years were treated with FNS or DHS for Garden III and IV fractures. Out of this geriatric cohort only eight (15.3%) patients showed mechanical failure. In the majority of cases with implant failure implant removal and arthroplasty was performed. In two young patients implant removal and reosteosynthesis was performed. In one geriatric patient due to the poor general condition only the implant was removed. Intraoperative factors of all 25 cut out cases are shown in Table 2.

**Table 2:** Parameters for the 25 cases with implant failure

Variable	DHS	FNS	*p* value
Cases with implant failure	11	14	*p* = 0.64
Age	66.6 ± 21.5	76.3 ± 13.3	*p* = 0.16
BMI	23.1 ± 2.5	24.0 ± 4.0	*p* = 0.62
AO classification			*p* = 0.58
31-B1	3	6	
31-B2	4	6	
31-B3	4	2	
Garden classification			*p* = 0.615
I	2	1	
II	3	4	
III	4	8	
IV	2	1	
Pauwels classification			*p* = 0.81
I	0	1	
II	5	4	
III	6	9	
Optimal blade position	4	7	***p* < 0.05**
Tip apex [mm]	22.0 ± 5.9	25.9 ± 8.3	*p* = 0.520
Secondary prothesis	9	13	
Implant removal	2	1	

*p* < 0.05 was used as cut off for bold significance

## Discussion

In this clinical study, the FNS proved to be an alternative in the treatment of hip fractures compared to DHS. Previous biomechanical studies showed comparable results for FNS compared with DHS and an increased stability compared to three screws [9] or Hansson Pins [10]. Chang-Ho et al. showed that center–center blade positioning and low tip apex distance will increase the stability of the FNS [11]. A finite element study from Fan et al. showed increased stability of the FNS compared to 3 screws but also a higher stability for the 2-hole plate in if the fracture angle exceeded 70° [12]. There are only a few clinical studies evaluating the clinical use of the FNS. Stassen et al. showed for 34 patients’ surgical complications in 23.5% [13]. Yan et al. evaluated 24 patients with FNS compared to 58 patients with CCS showing a surgical complications rate of 8.3% in the FNS group [14]. In the 47 patients treated with FNS in the study of Tang et al., 12% had surgical complications [15]. The surgical complication rate in this larger cohort was 13.3% and, therefore, within the range of the mentioned studies and lower compared to DHS group, despite that the mean age was considerably higher in our study. All mentioned studies reported cases with avascular necrosis, which did not occur in any patients of this study. This might be explained with the low time to surgery of 13.5 h. Compared to DHS the FNS group had a lower surgical complication rate. While cut out was slightly more often (FNS 12.4% vs. DHS 10.2%), there were considerably less cases with hematoma and no case of implant related infection in FNS group. With careful surgical planning, the FNS can be implanted through a 3–4 cm incision and is, therefore, more minimal invasive than DHS [16]. The incision size in the study of Tang et al. was 4 cm in FNS group and comparable to the incision of the CCS group. There was no significant difference between the groups with regard to cut outs, underlining the biomechanical comparisons that showed equal stability for both implants. The main influence to prevent mechanical failure is still the surgeon. Less than 50% of the patients with cut out of the FNS or DHS-blade had an optimal blade position. The relative risk for cut out significantly increased sevenfold for none optimal blade positioning. Despite that, multiple studies already showed an increased rate of cut out with blade position deviating from center–center and inferior–center [17–20] there were still around 88% of the patient had optimal blade positions in this study. This highlights the importance of the correct surgical technique regardless of the implant. The tip apex distance was no significant predictor of implant failure in this study. In both groups mean tip apex distance was less than 25 mm, what might have prevented further cut outs compared to the results of studies including more cases with tip apex distance higher than 25 mm [19, 20]. Tip apex distance was slightly less in FNS group. The authors used the described technique from Cha et al. to ensure low tip apex distance in all cases [21]. Cut outs in the FNS group occurred significantly more often in Pauwels III fractures. This might be explained with the usage of only 1-hole plates for the FNS. Yan et al. showed a lower biomechanical stability of the FNS when the fracture angle exceed 70° and only a 1-hole plate is used [14]. Therefore, the author suggests using 2-hole plates in Pauwels III fractures to prevent implant failure at the side of the plate (Fig. 1B). In both groups, patients between 20 and 98 years of age with Garden III/IV as well as Pauwels III fractures were included. Neither fracture classifications nor age showed a statistically significant influence on surgical complications and implant failure. While current guidelines recommend osteosynthesis only in undisplaced fractures in patients over 60 years [4] the authors also used it in Garden III and IV fractures. Overall 52 patients older than 60 years were treated with FNS or DHS for Garden III and IV fractures if the overall condition of the patients does not allow arthroplasty. Out of the geriatric cohort only eight patients showed mechanical failure. This highlights that both implants can be used even in older patients as an alternative to endoprosthesis for patients in poor overall condition. There is evidence that patients with severe comorbidities might profit from osteosynthesis compared to arthroplasty [22]. If mechanical failure was detected in X-ray controls and the patient presented with pain and impaired functionality of the hip revision surgery was performed in the majority of the cases with arthroplasty. Only two young patients were treated with reosteosynthesis.

Hip fractures treated with osteosynthesis have a risk of femoral neck shortening which can result in a difference of leg length. The FAITH trial showed comparable femoral neck shortening of DHS compared to CCS [8]. Femoral neck shortening was significantly less in the FNS group compared to the CCS group in the study of Tang et al. Compared to both studies the rate of femoral neck compression over 5 mm of the FNS in our study was comparable. Still, the DHS showed no difference in mean shortening or rate of shortening over 5 mm compared to the FNS in this study. Furthermore, Haider et al. showed that femoral neck shortening did not impair functional outcome in the majority of cases [23]. The FNS could be implanted with shorter operating time and less Hb-difference and the patients could be released home or into rehabilitation significantly earlier. Compared to the study of Yan et al. and Tang et al. the FNS was implanted 22 min and 16 min faster in our study, which might be explained by a higher case load of approximately 40 cases a year at our institution.

In summary, the "new" FNS is slightly better regarding operating time, Hb-difference and duration of hospital stay and can be used for all ages and fracture patterns. However, the most important factor is not the implant, but the correct surgical technique to prevent surgical complications.

## Conclusion

The FNS is a new strong treatment option for femoral neck fractures for patients of all ages. Compared to DHS it can be implanted faster and with less blood loss. Surgical complication rates are comparable, especially rate of cut out. However, most important is not a modern implant but a surgical technique respecting optimal blade positioning and tip apex distance less than 25 mm.
